# Organizational Changes to Thyroid Regulation in *Alligator mississippiensis*: Evidence for Predictive Adaptive Responses

**DOI:** 10.1371/journal.pone.0055515

**Published:** 2013-01-30

**Authors:** Ashley S. P. Boggs, Russell H. Lowers, Jessica A. Cloy-McCoy, Louis J. Guillette

**Affiliations:** 1 School of Natural Resources and Environment and Department of Biology, University of Florida, Gainesville, Florida, United States of America; 2 Department of Obstetrics and Gynecology, Medical University of South Carolina, and Hollings Marine Laboratory, Charleston, South Carolina, United States of America; 3 Innovative Health Applications, National Aeronautics and Space Administration, Kennedy Space Center, Florida, United States of America; University Claude Bernard Lyon 1, France

## Abstract

During embryonic development, organisms are sensitive to changes in thyroid hormone signaling which can reset the hypothalamic-pituitary-thyroid axis. It has been hypothesized that this developmental programming is a ‘predictive adaptive response’, a physiological adjustment in accordance with the embryonic environment that will best aid an individual's survival in a similar postnatal environment. When the embryonic environment is a poor predictor of the external environment, the developmental changes are no longer adaptive and can result in disease states. We predicted that endocrine disrupting chemicals (EDCs) and environmentally-based iodide imbalance could lead to developmental changes to the thyroid axis. To explore whether iodide or EDCs could alter developmental programming, we collected American alligator eggs from an estuarine environment with high iodide availability and elevated thyroid-specific EDCs, a freshwater environment contaminated with elevated agriculturally derived EDCs, and a reference freshwater environment. We then incubated them under identical conditions. We examined plasma thyroxine and triiodothyronine concentrations, thyroid gland histology, plasma inorganic iodide, and somatic growth at one week (before external nutrition) and ten months after hatching (on identical diets). Neonates from the estuarine environment were thyrotoxic, expressing follicular cell hyperplasia (p = 0.01) and elevated plasma triiodothyronine concentrations (p = 0.0006) closely tied to plasma iodide concentrations (p = 0.003). Neonates from the freshwater contaminated site were hypothyroid, expressing thyroid follicular cell hyperplasia (p = 0.01) and depressed plasma thyroxine concentrations (p = 0.008). Following a ten month growth period under identical conditions, thyroid histology (hyperplasia p = 0.04; colloid depletion p = 0.01) and somatic growth (body mass p<0.0001; length p = 0.02) remained altered among the contaminated sites. This work supports the hypothesis that embryonic EDC exposure or iodide imbalance could induce adult metabolic disease states, thereby stressing the need to consider the multiple environmental variables present during development.

## Introduction

Proper thyroid hormone signaling is necessary for metabolic regulation and growth. A poor embryonic environment that induces hyperthyroidism or hypothyroidism can lead to organizational (permanent) changes that greatly alter juvenile and adult physiology [1], [2]. These changes could be described as ‘predictive adaptive responses’ (PARs), prenatal alterations that maximize survival in a similar postnatal environment [3]. For example, a nutrient-poor embryonic environment is associated with small neonates that are metabolically adapted to low nutrient environments, which would be a ‘thrifty phenotype’ in a nutrient-poor external environment [4].

A possible mechanism for the thrifty phenotype is a resetting of the hypothalamic-pituitary-thyroid (HPT) axis through reduced pro-thyrotropin releasing hormone (TRH), gene expression in the neurons of the hypothalamus [5]. Briefly, the regulation of thyroid hormones is primarily under the control of the HPT axis. When plasma concentrations are reduced, TRH, produced by the hypothalamus, travels to the anterior pituitary to stimulate thyrotropin (TSH) release, which in turn stimulates thyroid hormone production and secretion from the thyroid gland [6]. Thyroid hormones are produced in two forms, thyroxine (T_4_), the more abundant but less active prohormone, and triiodothyronine (T_3_), the less abundant, more active hormone [6]. Thyroid hormones in circulation induce a negative feedback loop that halt pituitary TSH release [6], [7]. When thyroid hormones are mis-regulated, overstimulation by TSH can cause abnormalities of the thyroid gland such as reduction of the luminal colloid, follicular cell hyperplasia, and follicular cell hypertrophy [8]. Using the example above, reduced proTRH gene expression in the neurons of the hypothalamus could dampen TSH release, despite depressed plasma thyroid hormone concentrations [5]. In this manner, the sensitivity of the HPT-axis to reduced thyroid hormone concentrations is decreased and a reduced metabolism and growth rate are maintained to increase chances of survival in a nutrient poor postnatal environment.

However, resetting the sensitivity of the HPT-axis, while adaptive in similar prenatal and postnatal environments, could result in disrupted thyroid regulation if the embryonic environment is not predictive of the postnatal environment. Reduced nutrition during embryonic development, followed by increased neonatal nutrition has been linked to adult metabolic disorders, hyperphagia, and obesity [9], [10].

Though the HPT axis regulates many aspects of thyroid hormone homeostasis, environmental influences, such as iodide consumption and endocrine disruption, can alter thyroid hormone concentrations as well. Iodine is a limiting element in the production of thyroid hormones; however, iodide imbalance can lead to hyperthyroidism or hypothyroidism [11]. Further, endocrine disrupting chemicals (EDCs), which alter the synthesis, clearance, or binding of hormones, can alter thyroid hormone regulation. Exposures to EDCs such as polychlorinated biphenyls (PCBs) or polybrominated diphenyl ethers (PBDEs), are associated with hypothyroidism in mammals and birds [12], [13], [14]. Because EDCs and iodide can alter thyroid hormone concentrations, which could developmentally reset the metabolic controls of the HPT-axis, it is possible that prenatal EDC exposure or iodide imbalance could developmentally alter thyroid regulation, resulting in altered growth and thyroid disorders in later life stages through mismatched PARs.

The aim of this experiment was to explore whether wild American alligators prenatally exposed to different EDCs and iodide concentrations via the nutrients and contaminants maternally encountered and sequestered into the egg, altered thyroidal endpoints and growth later in life. The American alligator has been studied as a model for endocrine disruption for nearly 20 years [15] and is also an obligate-freshwater predator which is also found in iodide-rich estuarine environments [16]. That is, alligators do not have salt secreting glands, like their phylogenetic relatives, the crocodiles; thus, they must have a freshwater source to offload excess salt via the production of urine [17]. Because alligators evolved in freshwater environments, iodide imbalance could occur among coastal populations with increased exposure to environmental iodide, thereby altering thyroid hormone concentrations and growth. Though the relationship between thyroid hormones and growth have not been directly studied in alligators, other reptiles and ectotherms show significant reductions in growth with reduced thyroid hormone concentrations [18], [19]. Therefore, we hypothesized three different outcomes from this experiment: 1) that neonatal alligators from an iodine-rich estuarine environment with contaminants known to be thyroid antagonists would have different thyroidal outcomes when compared to neonatal alligators from contaminated or reference freshwater sites; 2) that neonatal alligators from a freshwater site, highly contaminated with EDCs, would display depressed plasma thyroid hormone concentrations, increased thyroid follicular cell hyperplasia and hypertrophy, and altered growth compared to neonatal alligators from a reference freshwater site and from a contaminated estuarine site; 3) while many of these differences would be remediated through a controlled diet and clean freshwater, developmental programming during embryonic development would have lasting effects on thyroid regulation and growth. Our aim was achieved through observations of iodide-correlated plasma T_3_ concentrations among the estuarine population, and hypothyroidism among the freshwater EDC-exposed population in comparison to the reference population. These conditions were not completely ameliorated by clean freshwater and a controlled diet, as lasting differences in growth rates and thyroid histology were noted.

## Methods

All animal collections and experiments were conducted ethically with the approval of the State of Florida Fish and Wildlife Conservation Commission (permit AMP08X-01), United States Department of the Interior Fish and Wildlife Service (permits 08-002 and 2006 SUP 55), and the University of Florida Institutional Animal Care and Use Committee (project 201005319). All necropsies were performed after sodium-pentobarbital euthanasia to minimize suffering.

### Study Sites

Lake Woodruff National Wildlife Refuge (LWNWR) (lat. 29°06′N, long. 81°25′W) is a freshwater body on the St. John's River in central Florida. There is relatively little surrounding industry and human development making the refuge our reference site for low EDC contamination. The resident population of alligators has been studied for nearly 25 years (for characteristics of this lake, see Guillette et al. 1999 [20]).

Lake Apopka (AP) (lat. 28°40′N, long. 81°38′W) is a freshwater lake in central Florida which has a long history of anthropogenic contamination from agricultural pesticide runoff including dichlorodiphenylchloroethane (DDT) and its metabolites. Following a major spill in 1980, egg viability decreased dramatically and sexual development of resident alligators was altered [20], [21], [22]. This site also contains elevated PCB contamination [23], [24] suggesting alligators from this site could have altered thyroidal regulation. It is the site of ongoing remediation for nutrient and pesticide contamination.

Merritt Island National Wildlife Refuge (MINWR) (lat. 28°59′ N, long 80°61′ W) is located on a barrier island off the east coast of Florida containing several freshwater lakes and impoundments. Resident alligators are commonly found in the freshwater and saltwater environments. MINWR is also the location of NASA's Kennedy Space Center and has the potential for contamination from local aerospace activities. Ongoing studies by our research group have documented elevated concentrations of PCBs and variable concentrations of PBDEs in the muscle of juvenile alligators from MINWR that are comparable to those concentrations found at AP (Isobe et al. unpublished data). For additional information of this site, see Boggs et al. [16].

### Neonatal Sample Collection

Entire clutches (AP: N = 8; MINWR: N = 5; LWNWR: N = 6) were collected from our three study sites prior to sex determination. Following transport to the laboratory, eggs were candled for viability and one embryo was staged for development of the clutch. Eggs were then numbered with pencil, repacked in autoclave sterilized sphagnum moss, and incubated, one clutch per bin, at 30°C to produce 100% female offspring.

Upon hatching, neonates were tagged with a Monel web tag and morphometric measurements (body mass and snout-to-vent length (SVL)) were collected. Neonatal alligators have a large residual yolk mass in their gut and do not require feeding for seven to ten days, depending on ambient temperature, as they digest the yolk mass. Therefore, animals were allocated to large growth tanks according to clutch, but were not fed initially to exclude external nutritional influences.

One week post hatching, a subset of neonates randomized by weight (AP: N = 60; MINWR: N = 35; LWNWR: N = 50) was euthanized and necropsied. A blood sample was obtained from the postcranial vertebral sinus and collected in a (lithium) heparinized Vacutainer® prior to euthanization. Centrifuged plasma was aliquoted and frozen at −20°C for T_4_ and T_3_ analysis. The entire thyroid gland was fixed in Bouin's fixative and embedded in paraffin wax for histological examination.

### Growth Experimental Design

After selected neonates were sampled, the remaining animals were pit tagged (Biomark HPT8) in the side of the tail at the fourth whorl and were resorted into tanks according to weight. An assortment of animals from all clutches from all lakes were housed in each large grow-out tank and fed Mazuri® crocodilian diet (5MG1) *ad libitum*. Tanks were cleaned and fresh water provided daily. Acclimation was allowed to occur for five months to allow for removal of all the neonates sampled for the first experiment. After acclimation, weight and SVL measurements were recorded every two weeks. Animals were resorted by weight and total tank mass every month to reduce competition and crowding.

Once the first quartile of the animals reached one kilogram in weight, the remaining animals in the growth study (AP: N = 94; MINWR: N = 52; and LWNWR: N = 65) were necropsied. Each clutch had a minimum of ten individuals sampled except for one clutch from AP (clutch 8) which had only eight individuals due to mortality. Necropsies on juveniles were identical to the methods described above for neonates, except the thyroid gland was halved for fixation to ensure perfusion.

### Histological Analysis of the Thyroid Gland

Whole thyroid glands from a subset of neonates (AP: N = 16; MINWR: N = 11; LWNWR: N = 13) and one lobe of a subset of juvenile alligators (N = 18 from each site) were selected and matched by gland size. Thyroids were sectioned at 6 µm and stained with either modified Masson's trichrome or hematoxylin and eosin (H&E).

**Figure 1:**
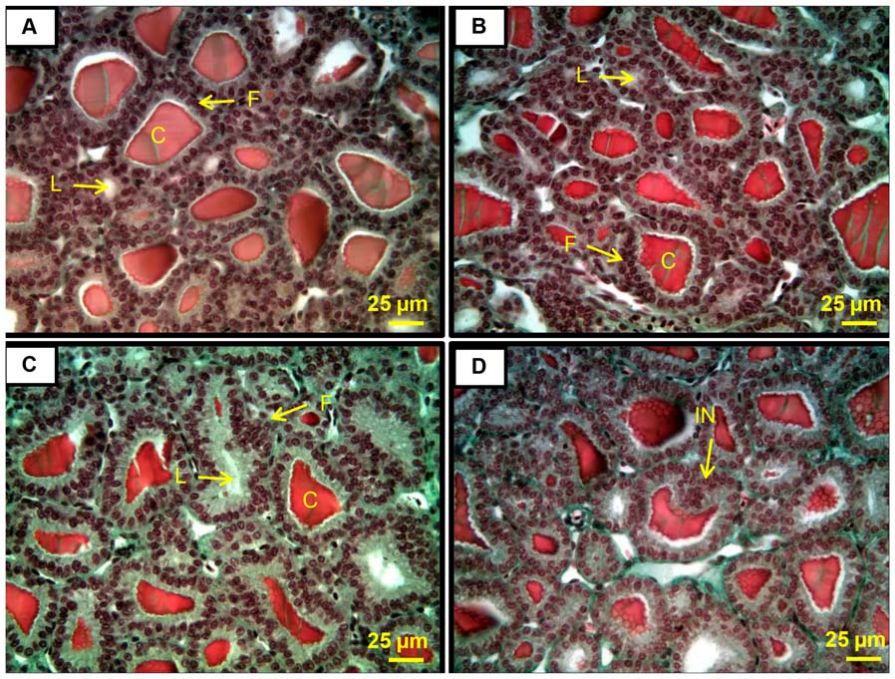
Thyroid gland histology from neonatal alligators. Thyroids were rated from 0 to 3 according to hyperplasia severity, hyperplasia area, and hypertrophy. A. All ratings of 1 (mild). B. All ratings of 2 (moderate). C. All ratings of 3 (severe). D. An example of follicular cell involution. F  =  follicular cell, C  =  colloid, L  =  lumen, IN  =  involution.

Analysis was conducted without knowledge of the site of origin of the animal following OSCP/EPA guidelines [25]. Briefly, area of colloid depletion, follicular cell hyperplasia, and follicular cell hypertrophy were given an area rating of 0 through 3 (0 = <20%, 1 = 21-50%, 2 = 51-80%, 3 = >81%) [26]. Follicular cell hyperplasia was also analyzed by counting the highest number of cell layers per follicle as a percentage of the total number of follicles. Severity of hyperplasia and the greatest severity of hyperplasia were rated from 0 to 3 using the rating system of Hooth et al. (0 =  one cell layer of squamous to cuboidal follicular cells, 1 =  at least two follicles with two to three cell layers, 2 =  more than two follicles with more than three cell layers, 3 =  significant infolding, branching, and microfollicular formations) [27]. Examples of the rating system are displayed in photomicrographs (Figure 1).

### Proximity Scintillation Radioimmunoassay

As described above, plasma samples were collected from the neonates prior to euthanization. Plasma samples were also collected from the animals in the growth study one month after hatching and at termination of the experiment (approximately ten months). Plasma total T_4_ and T_3_ concentrations were determined using validated proximity scintillation RIA techniques described in Boggs et al. [16]. Briefly, 96-well, solid-phase, protein-A flashplates plus (Perkin Elmer SMP-102) were coated with antibody (Fitzgerald Industries 20-TS45 for T_3_ and 20-TS40 for T_4_) and incubated overnight. Samples were prepared in phosphate buffered saline with 0.1% gelatin (PBSG; 0.1 M, pH 7.0). ^125^I-labeled steroids for T_3_ or T_4_ (Perkin Elmer; catalog number NEX110 or NEX111 respectively) were added at approximately 12,000 cpm and allowed to incubate for three hours until scanned using the Microbeta 1450 Trilux counter on the ^125^I channel.

### Plasma Inorganic Iodide Concentrations

A subset of plasma samples obtained for hormone analysis were selected for plasma inorganic iodide (PII) concentration determination by inductively coupled plasma mass spectrometry (ICP-MS). Due to sample limitations, one sample, which best represented the mean size from each clutch, was chosen for both the neonatal and juvenile groups. The specifics and validation of the ICP-MS measurement of PII can be found in Boggs et al. [16]. Briefly, samples were diluted in 10% absolute ethanol and thyroid hormone containing proteins measuring 3000 kDa were removed through gravity filtration using Amicon Ultra-4 centrifugal filter units (Milipore UFC800324). Samples were reconstituted in Mili-Q water and a Dionex ICS-3000 ion chromatography (IC) system was used for the separation of iodine species using a 50 mM nitric acid mobile phase at 1.0 ml/min with a Dionex IonPac AS11 (4 mm–250 mm) analytical column (052960), followed by a Thermo Elemental X7 ICP-MS (Peltier). Validations using standard reference material 3668 as well as a spike retrieval in alligator plasma were confirmed for this sample set. Results were drift corrected through bracketed High Purity Standards (ICII-M, 1000 µg/ml ±0.5% µg/ml).

### Statistical Analysis

All statistics were conducted with JMP 8.0. Measurements at hatching and growth rates of SVL and body mass were compared across sites by the methods described by Webb et. al [28]. Briefly, the equation for growth rate is 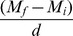



where *M_f_* is the final measurement, *M_i_* is the initial measurement, and *d* is the number of days between measurements. Growth rates were then analyzed by linear regression with the mean measurement of interest as the independent variable [28]. This is the most appropriate model for crocodilians between 15 and 80 cm SVL because during this time, the relationship between growth rate and size is linearized using this model [28], [29]. Mean measurement of interest was calculated by the equation
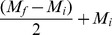



to determine differences in growth patterns among sites of origin. Differences among sites were tested by ANCOVA with the mean measurement as the covariate.

Histological data were analyzed by two methods. Hyperplasia cell count data were checked for normality. If the data were not normally distributed, a square root transformation was used. Homogeneity of variance was checked in all procedures by the Brown-Forsythe test prior to all procedures testing significance. If all assumptions were met, ANOVAs were conducted among sites followed by a post-hoc Tukey test if p ≤0.05. Area and severity scores were analyzed among sites using the Kruskal-Wallis (KW). In all KW analyses, if p ≤0.05, multiple t-tests with a Bonferroni correction were conducted to determine differences among sites.

Plasma thyroid hormone concentrations were checked for normality and log transformed if necessary. If variance was not equal among groups, a KW test was conducted. Otherwise, differences in plasma thyroid hormone concentrations were determined by ANOVA.

PII concentrations were analyzed by ANOVA to determine a significant difference in iodide concentrations among animals from different sites. Correlations were conducted between site-specific iodide concentrations and plasma T_3_ and T_4_ concentrations.

## Results

### Histological Assessment

**Figure 2:**
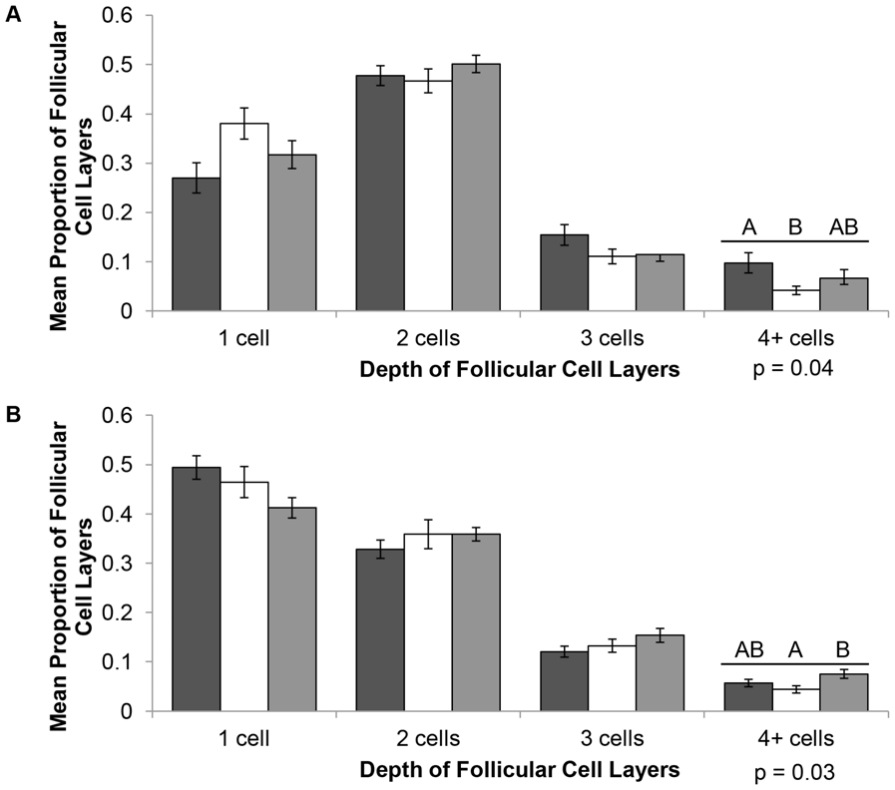
Thyroid follicular cell hyperplasia. Mean proportions of follicular cell layers per thyroid gland of alligators from three sites in Florida. Merritt Island  =  black, Lake Woodruff  =  white, Lake Apopka  =  gray. A. Neonatal alligators. (AP: N = 16; MINWR: N = 11; LWNWR: N = 13) B. Juvenile alligators (N = 18 for all sites). Error bars are ± SEM. Letters represent significant differences among sites within follicular cell layer classifications.

Among all sites, neonatal follicular cell layers were significantly different for the percentage of follicles with four or more cell layers (p = 0.04; Figure 2A). Neonates from MINWR had significantly more follicles with four or more cell layers than neonates from the reference (WO). The area of hyperplasia and the hyperplasia severity were significantly different among sites (p = 0.01 and 0.001, respectively). The area of hyperplasia and severity of hyperplasia were greater among AP and MINWR neonates compared to LWNWR neonates. The area of hypertrophy, the greatest severity of hyperplasia, and area of colloid depletion were not significantly different among sites (Table 1).

**Table 1:** Mean classification for thyroid histological markers of American alligators.

	Neonate			Juvenile		
Site	MINWR	LWNWR	AP	MINWR	LWNWR	AP
Hypertrophy	2.73 (0.27)	2.54 (0.24)	2.63 (0.16)	2.67 (0.49)	2.56 (0.62)	2.83 (0.51)
Hyperplasia	2.27 (0.14)*	1.69 (0.13)	2.31 (0.15)*	1.50 (0.12)	1.44 (0.12)	1.83 (0.09)
Severity of Hyperplasia	2.64 (0.15)*	1.38 (0.18)	2.13 (0.20)*	1.94 (0.17)	1.61 (0.14)	2.28 (0.20)*
Greatest Severity of Hyperplasia	2.82 (0.12)	2.69 (0.13)	2.56 (0.13)	2.39 (0.12)	2.11 (0.14)	2.61 (0.12)*
Colloid Depletion	2.27 (0.24)	1.54 (0.29)	1.63 (0.18)	0.389 (0.12)*	0.00 (0.00)	0.389 (0.12)*

* Indicates statistically different from the reference (LWNWR)

Number in parenthesis is standard error

Juvenile alligators from AP had more follicles with four or more cell layers (Figure 2B) and greater severity of hyperplasia compared to LWNWR juveniles (p = 0.03 and p = 0.04 respectively). Colloid depletion was significantly greater among AP and MINWR juveniles compared to LWNWR juveniles (p = 0.01). The greatest severity of hyperplasia was significantly higher among AP juveniles compared to LWNWR juveniles (p = 0.03). Area of hyperplasia and area of hypertrophy were not significantly different among sites (Table 1).

### Plasma Thyroxine and Triiodothyronine Concentrations

**Figure 3:**
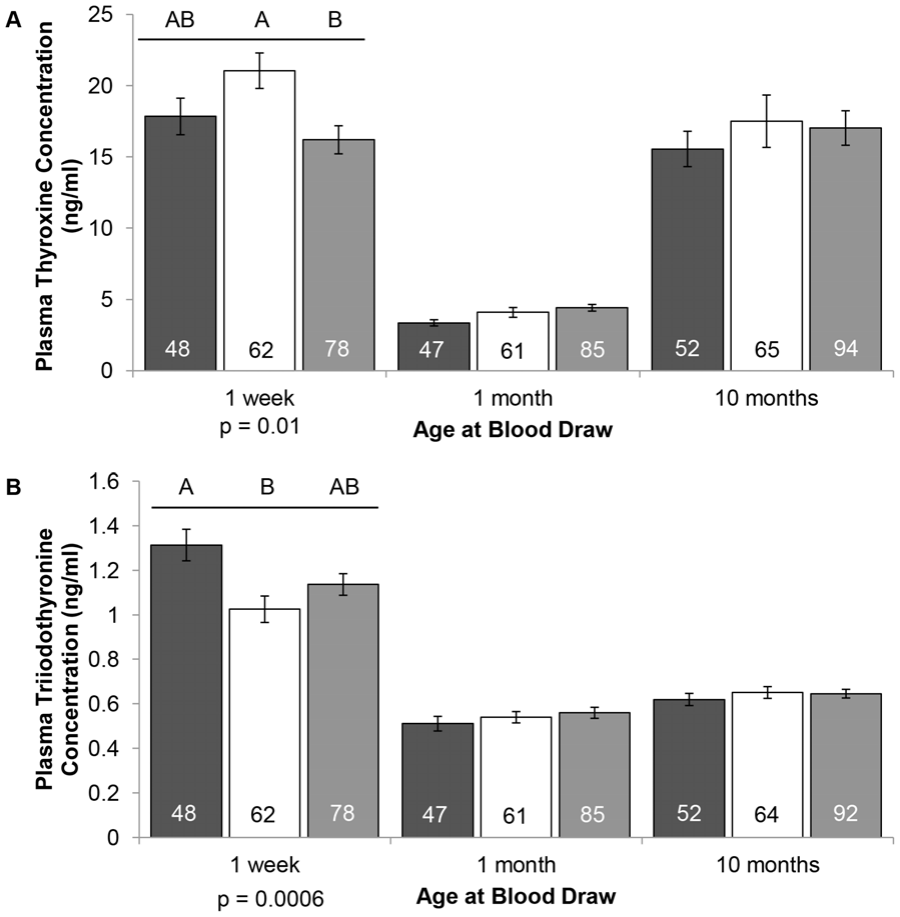
Plasma thyroid hormone concentrations from different sites and life stages of American alligators from Florida. Merritt Island  =  black, Lake Woodruff  =  white, Lake Apopka  =  gray. Letters represent statistical differences among sites within age groups. Numbers within bars represent sample size. Error bars are ± SE. A. Mean plasma thyroxine concentrations. B. Mean plasma triiodothyronine concentrations.

Neonatal alligators from AP had significantly depressed plasma T_4_ concentrations when compared to LWNWR neonates (p = 0.01; Figure 3A). Neonatal plasma T_3_ concentrations at MINWR were significantly elevated compared to LWNWR neonates (p = 0.0006; Figure 3B).

Among juvenile alligators raised in captivity, plasma T_4_ concentrations of one-month old alligators were significantly elevated in AP animals compared to MINWR animals (p = 0.02; Figure 3A), but plasma T_3_ concentrations were not significantly different among sites (Figure 3B). After 10 months there were no significant differences in plasma T_4_ and T_3_ concentrations among sites (Figures 3A and 3B).

### Plasma Inorganic Iodide Concentrations

**Figure 4:**
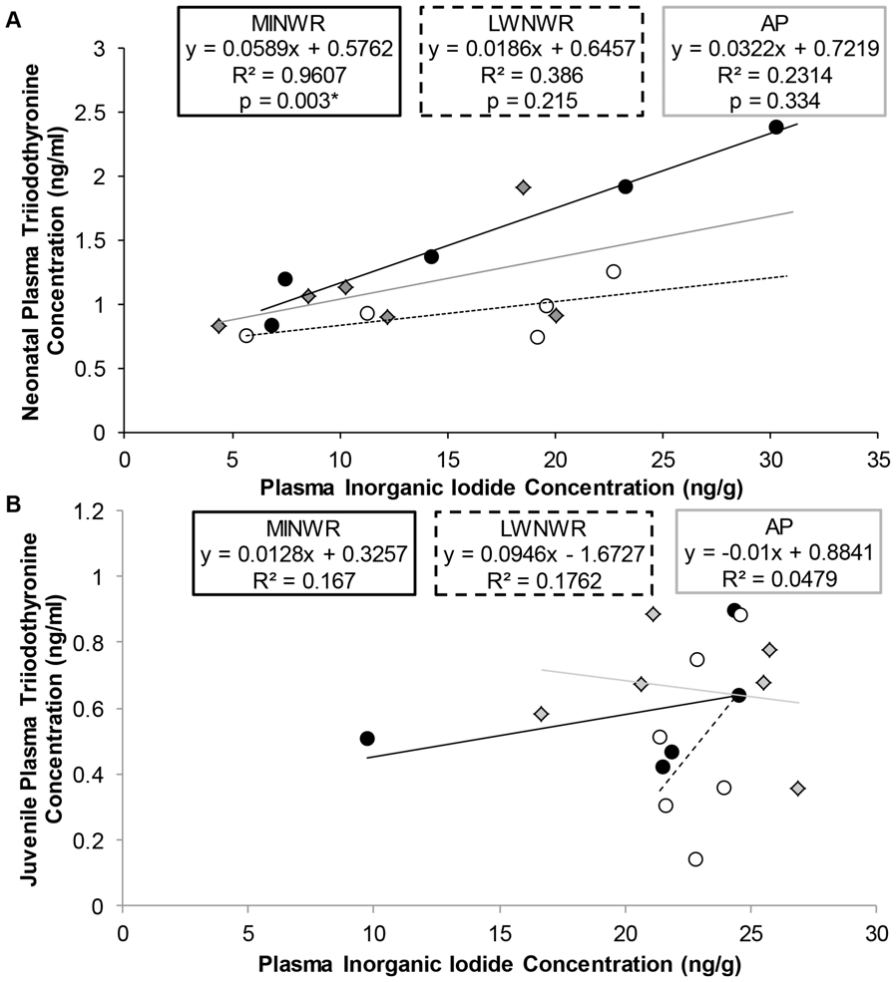
Relationship between plasma triiodothyronine concentrations and plasma inorganic iodide concentrations of alligators. Merritt Island  =  black circles and black line, Lake Woodruff  =  white circles and dotted line, Lake Apopka  =  gray diamonds and gray line. A. Neonates B. Juveniles.

Analysis of PII indicated no significant difference among neonates or juveniles according to site of origin. Neonatal plasma T_4_ concentrations did not correlate with PII concentrations. Plasma T_3_ concentrations among neonates from MINWR were highly positively correlated with neonatal PII concentrations (p = 0.003 and r^2^ = 0.961) but were not significantly correlated among neonates from LWNWR or AP (Figure 4A). Juvenile plasma thyroid hormone concentrations were not significantly correlated with juvenile PII concentrations (Figure 4B).

### Morphometrics and Growth Rates

**Figure 5:**
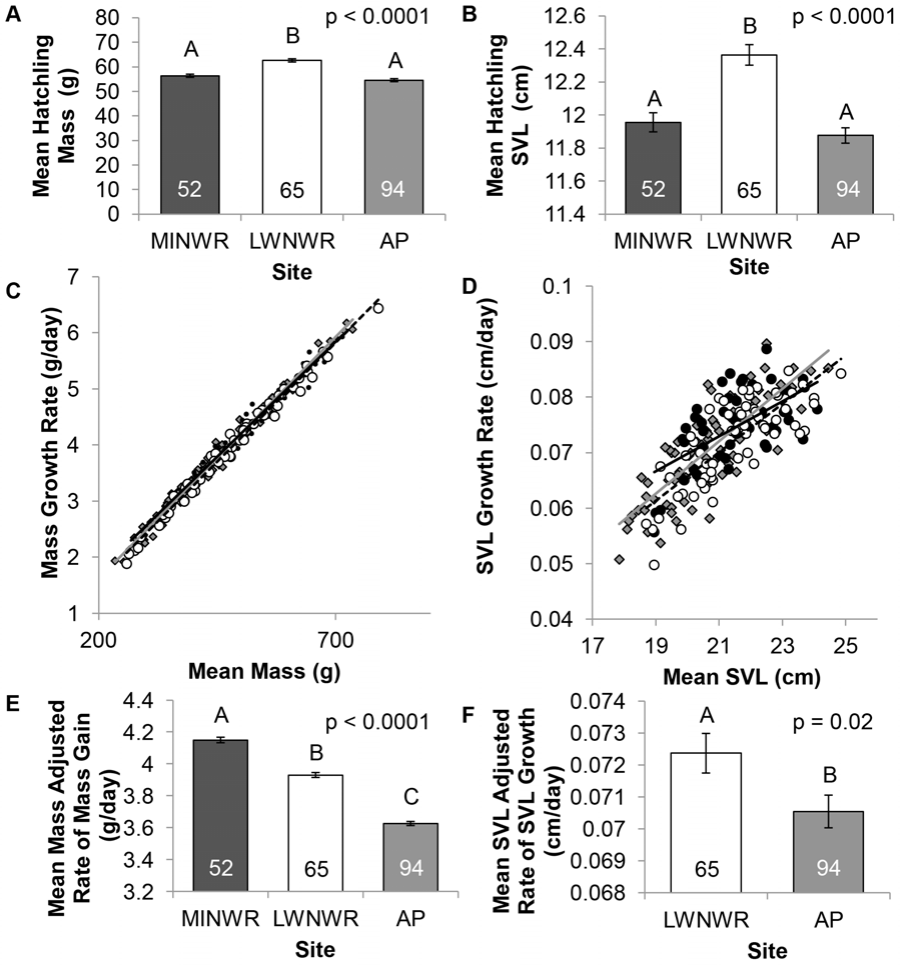
Growth comparisons of alligators from three sites in Florida. Merritt Island  =  black circles and black line, Lake Woodruff  =  white circles and dotted line, Lake Apopka  =  gray diamonds and gray line. Letters represent significant differences among sites. Numbers within bars represent sample size. Error bars are ± SE. A. Snouth-vent length (SVL) measurements at hatching. B. Body mass measurements at hatching. C. Linear regression of body mass growth rates and mean body mass of captive reared juvenile alligators from three central Florida sites. R^2^ values were 0.997 for Lake Apopka, 0.993 for Merritt Island, and 0.997 for Lake Woodruff. D. Linear regression of snout-to-vent- length (SVL) growth rates and mean SVL mass of captive reared juvenile alligators from three central Florida sites. R^2^ values were 0.705 for Lake Apopka, 0.383 for Merritt Island, and 0.623 for Lake Woodruff respectively. E. Analysis of mean body mass adjusted rate of body mass gain in captive juvenile alligators from three different sites. F. ANCOVA of mean SVL adjusted rate of SVL gain in captive juvenile alligators from Lake Woodruff and Lake Apopka.

Hatchlings from LWNWR had greater body mass and larger SVLs than those from AP or MINWR (p<0.0001 for both; Figure 5A and 5B). Under optimal growth conditions, body weight gain models showed that animals gained weight faster with increasing mean weight (Figure 5C). Likewise, juvenile SVL growth models indicate that SVL growth rates increased with increasing mean SVL (Figure 5D). MINWR juveniles had a depressed SVL growth rate model compared to AP and LWNWR juveniles.

ANCOVAs among the three sites were conducted on rate of body mass gain and rate of SVL gain among sites with respectively mean body mass or mean SVL as the covariate. However, because mean body mass and mean SVL were significantly different among sites, thereby violating one of the conditions for a suitable covariate, the difference of the mean body mass by site and the individual mean body mass was calculated to serve as the covariate. Compared to the reference juveniles from LWNWR, juveniles from MINWR gained mass at an accelerated rate while juveniles from AP gained mass at a depressed rate (p<0.0001; Figure 5E). An ANCOVA was conducted on SVL growth rates, but data from MINWR animals was excluded because of the difference in the relationship of mean SVL and SVL growth rates when compared to AP and LWNWR. Results showed that juveniles from AP grew in length at a slower rate than juveniles from LWNWR (p = 0.02; Figure 5F). There were no significant correlations between growth rates and plasma thyroid hormone concentrations.

## Discussion

Our data support the hypothesis that embryonic environmental conditions can alter the morphology and physiology of the thyroid gland. In this study, we found that embryonic environmental conditions induced thyrotoxic changes in neonatal alligators from MINWR and hypothyroidism in neonatal alligators from AP. Prolonged captivity under ideal conditions did not resolve all thyroid endpoints.

Alligators from the estuarine environment of MINWR displayed numerous hyperthyroid markers that were closely associated with PII concentrations. Before the introduction of external nutrition, MINWR alligators displayed elevated plasma T_3_ concentrations and severe thyroid follicular cell hyperplasia compared to the reference. This thyroid gland morphology is indicative of overstimulation of the thyroid gland [26]. In neonatal humans, elevated T_3_ concentrations and normal T_4_ concentrations accompanied by thyroid gland hyperplasia is often diagnosed as T_3_ thyrotoxicosis, a disease attributed to elevated thyroid hormone ingestion or excess iodide exposure [30], [31]. Therefore, there are two hypotheses as to the induction of T_3_ thyrotoxicosis in neonatal alligators from MINWR. When compared to freshwater females, reproductively active female alligators from MINWR could be partitioning elevated thyroid hormone concentrations into the egg yolk. This hypothesis would require elevated thyroid hormone concentrations among wild MINWR alligators compared to freshwater alligators, which has been described previously by our lab [16]. Although thyroid hormone concentrations have not been measured in the alligator egg, there is evidence of maternal partitioning of thyroid hormones into the yolk in accordance with maternal thyroidal status in another archosaur [32], [33]. An alternative hypothesis for neonatal thyrotoxicosis among the MINWR neonates is increased maternal partitioning of iodide, not thyroid hormone, into the eggs. In this study, 95% of the variance in plasma T_3_ concentrations of neonatal alligators from MINWR could be explained by PII concentrations, whereas there was no correlation between PII and thyroid hormones among neonates from the freshwater sites. The next rational step is to measure thyroid hormones and iodide concentrations of maternal alligators, their offspring, and the egg yolk to clarify the relationship of maternal contribution to neonatal thyrotoxicosis in the American alligator.

After 10 months of controlled ambient heat, regulated diet, and clean fresh water, most of the hyperthyroid and thyrotoxic markers were ameliorated. Plasma T_4_ and T_3_ concentrations as well as PII concentrations were similar to those of the reference. Also, MINWR juveniles no longer displayed the correlation between PII and T_3_ concentrations seen in neonates. This return to euthyroidism is similar in infants with neonatal thyrotoxicosis which usually ameliorates within 48 weeks of age [34]. However, histological examination of the thyroid gland of juveniles from MINWR in this experiment displayed a reduction in colloid suggesting a reduction of thyroid hormone production or storage. In rats, neonatal hyperthyroidism has been associated with adult hypothyroidism [35]. This has been linked to permanent alteration of HPT-axis sensitivity from overstimulation during development [1], [36], [37]. Therefore, MINWR alligators could become hypothyroid from HPT-axis insensitivity due to neonatal thyrotoxicosis. However, the HPT-axis of the alligator has not been described and additional experiments are necessary to test this hypothesis.

Analysis of the development of alligators from an environment with agriculturally derived EDCs provided some novel insight into reptilian thyroid disruption. Neonates from AP given no external nutrition displayed a number of hypothyroid markers including depressed plasma T_4_ concentrations and increased thyroid follicular cell hyperplasia. Previous studies on wild juvenile alligators from AP have reported altered thyroid hormone concentrations compared to wild juvenile alligators from LWNWR [38], [39]. Additionally, PII concentrations of neonates from this freshwater environment, were not significantly correlated with plasma thyroid hormone concentrations. Therefore, we hypothesized that hypothyroidism observed in this population was likely from EDC exposure to thyroid disrupting contaminants that have been previously detected in eggs from this site [24].

After 10 months of captivity, AP juveniles had normal plasma thyroid hormone concentrations, but thyroid gland hyperplasia and colloid depletion among the AP alligators was not resolved. If the thyroid gland is stimulated to produce more follicular cells and store less thyroid hormones, plasma thyroid hormone concentrations could be maintained at normal levels despite persistent thyroidal disruption. This suggests that given a more stochastic environment such as one experienced in the wild, these animals could revert to a hypothyroid condition.

By interpreting the growth results in relation to the thyroid endpoints from this study, we can hypothesize that elevated iodide or thyroid hormones, likely in the yolk due to an estuarine maternal diet, among the alligators from MINWR, induced embryonic programming in a manner inconsistent with the freshwater, nutrient-rich, postnatal environment of captivity. We found that neonates from MINWR and AP were significantly smaller both in mass and length at hatching, indicating a poor embryonic environment compared to their LWNWR counterparts. After *ad libitum* food availability, though MINWR alligators rapidly gained weight, they did not increase in length in the same manner as AP or LWNWR alligators. Dampened SVL growth rates among the MINWR alligators could be related to iodide influenced elevated T_3_ concentrations during embryonic development and an altered HPT axis [40], [41] [35]. MINWR alligators could have reduced sensitivity of the HPT axis as a predictive adaptive response to elevated embryonic iodide exposure as a means to reduce the effects of elevated iodide in the postnatal environment at MINWR. In the mismatched, freshwater environment of this experiment, it is possible that MINWR alligators could have a reduced metabolism due to the resetting of the HPT axis leading to increases in body mass without similar increases in length. However, it should be noted that adaptive responses in a postnatal environment with elevated iodide were not tested and further experiments are required.

In contrast, the reduced growth rates in mass and length of AP alligators could be lasting effects on thyroid physiology due to yolk contaminants derived from the maternal diet. In this study, if PAR programming was induced by EDCs, theoretically, the energetic cost of metabolizing contaminants could shift the dynamic energy budget away from growth and toward toxicant metabolism to aid survival in an EDC contaminated postnatal environment [42]. Once EDC exposure is removed, one would expect faster growth rates due to the accelerated metabolic status generated by PAR programming. This accelerated growth pattern was described by Moore et al. in neonates from AP raised under similar conditions; however, Moore et al. also described a halt to the accelerated mass gain between four and five months of age [43]. This current study, which focused on growth solely after 5 months of age, also showed a reduced growth rate among the juvenile AP alligators. One possible hypothesis for this delayed reduction in growth rates could be that the body burden of persistent EDCs in AP alligators could continue to influence growth. EDC metabolites that have different metabolic effects than the original parent compounds could be present in higher concentrations after 10 months without additional EDC exposure. Thus, the embryonic/neonatal contaminant milieu may not be identical to the juvenile contaminant milieu leading to different growth rates at different life stages, but further experiments are required to test this hypothesis.

This study serves to highlight the necessity of considering multiple environmental influences during thyroidal developmental programming and regulation. While research on the effects of EDCs on metabolism is increasing, the influence of diet and nutrition are beginning to be recognized as major influences that alter the outcomes of EDC exposure. This study demonstrates that developmental programming can be influenced by multiple environmental factors including nutrient quality and availability.
